# Assessment of Knowledge, Attitude, and Practice of Obstetricians and Gynecologists Toward Off-Label Medicine Use in Female Reproductive Health Issues

**DOI:** 10.3389/fpubh.2022.829339

**Published:** 2022-03-24

**Authors:** Sadia Shakeel, Wajiha Iffat, Ambreen Qamar, Shagufta Nesar, Fareeha Butt, Sobia Naseem Siddiqui, Hina Rehman, Anees ur Rehman

**Affiliations:** ^1^Department of Pharmacy Practice, Faculty of Pharmaceutical Sciences, Dow College of Pharmacy, Dow University of Health Sciences, Karachi, Pakistan; ^2^Department of Pharmaceutics, Faculty of Pharmaceutical Sciences, Dow College of Pharmacy, Dow University of Health Sciences, Karachi, Pakistan; ^3^Department of Physiology, Dr. Ishrat Ul Ebad Khan Institute of Oral Health Sciences, Dow University of Health Sciences, Karachi, Pakistan; ^4^Jinnah College of Pharmacy, Sohail University, Karachi, Pakistan; ^5^Department of Physiology, Dow Medical College, Dow University of Health Sciences, Karachi, Pakistan; ^6^Department of Pharmacy Practice, Institute of Pharmaceutical Sciences, Jinnah Sind Medical University, Karachi, Pakistan; ^7^Department of Pharmacy Practice, Faculty of Pharmacy, Bahauddin Zakariya University Multan, Multan, Pakistan

**Keywords:** off-label prescribing, female reproductive health, gynecologists/obstetricians, Pakistan, knowledge

## Abstract

**Background:**

Off-label medication usage (OLMU) is prevalent in the treatment of various diseases, including female reproductive health issues (FRHIs). However, there is a paucity of literature on the perspective of health professionals on this subject. The purpose of the current study was to assess gynecologists/obstetricians' knowledge, attitude and practice toward OLMU in the treatment of FRHIs.

**Methods:**

The current cross-sectional study was conducted in September and October 2021, at five tertiary care hospitals (two public and three private sector), different clinics and maternity homes in a metropolitan city of Karachi, Pakistan. The target population was gynecologists, obstetricians and physicians/residents working in the ob/gyn department in various hospitals and clinical settings of Karachi.

**Results:**

The overall response rate was 77.1%. The mean age of the study respondents was 36.1 ± 7.7 years; *n* = 85 (55.9%) respondents were working in primary patient care. The majorly reported OLMU by the respondents were clomiphene citrate in unexplained infertility (*n* = 66; 43.4%), metformin to improve cycle regularity in females with polycystic ovary syndrome (PCOS) (*n* = 59; 38.8%) and letrozole to induce ovulation (*n* = 31; 20.4%). The majorly stated categories of OLMU were at a different dose (*n* = 95; 62.5%) and at different indications than approved to treat (*n* = 89; 58.5%). It was reported by the majority of the respondents (*n* = 95; 62.5%) that they do not follow any guidelines or regulations for OLMU in their work setting; however, the response was statistically varied with the working organization (CI 2.14–2.93; *p* = 0.037) and practice area (CI 2.85–4.32; *p* = 0.0001) of respondents.

**Conclusions:**

The present study revealed that the respondents were well-familiar with the practice of OLMU in the treatment of FRHIs. They expressed their concerns about decreasing such practices by being involved in collective decision-making procedures, and they were inclined to accept initiatives aimed at ensuring drug safety in patients.

## Introduction

The reproductive health of a woman is vital to her overall wellbeing. Reproductive health is described as a condition of physical, emotional, mental, and social wellbeing in all aspects of reproduction, rather than simply the absence of sickness, malfunction, or infirmity (1). Menstrual health, which marks the beginning of reproductive health, and menopause, which marks the end of reproductive health, are two frequently overlooked aspects. Likewise, female infertility affects millions of couples worldwide, and there are several drugs available to help with conception (1). In general, infertility is defined as the inability to conceive (get pregnant) after 1 year (or more) of unprotected sex. Since women's fertility declines progressively with age, some providers analyze and treat women aged 35 and above after 6 months of unprotected sex (1). Fertility therapies include hormone and ovulation-regulating medicines, occasionally combined with minor surgical interventions, and are evaluated by the Food and Drug Administration (FDA) to get a license for a specific mode of application. Many of the medications given to treat female infertility are used off-label, which means they have not passed all of the tests required for approval for a specific purpose (2).

Off-label medication usage (OLMU) is defined as prescribing medicines in a way that contradicts product usage information issued by regulatory authorities. OLMU can be grouped into several categories, including unapproved indications, use in a specific demographic, administration by an unauthorized method, and administration at a dose not mentioned in the FDA-approved label (2). OLMU has increased significantly across all medical specialties in preceding decades because it allows the prescribers to employ novel treatment alternatives based on the most recent data. The majority of medications used to treat FRHIs are used off-label (3). Misoprostol can be used to treat both early (miscarriage) and late pregnancy loss. Its extensive usage for the treatment of first-trimester miscarriage is strongly supported by high-quality evidence from throughout the world (4). Metformin is used therapeutically off-label in the treatment of hirsutism, acne, and insulin resistance in PCOS, despite conflicting data regarding anti-androgenic properties. Metformin can also be taken alone or in conjunction with clomiphene citrate to enhance ovulation in women with PCOS (3). Off-label usage of letrozole for ovarian stimulation and ovulation induction in anovulatory and unexplained infertility. Bromocriptine is a dopamine agonist that is used off-label in the treatment of pituitary origin infertility and ovarian hyperstimulation syndrome (OHSS) (3).

The legislation governing the use of off-label drugs has not been unified globally. The suitability of OLMU stays a matter of discussion, due to their susceptibility regarding the proven medical benefits and probable toxicities, little or no evidence to assist in scientific decision-making, increased treatment expenses for patients and ethical concerns (5). National legislation, rules, or recommendations regulating OLMU have been created in certain developed nations, such as the United Kingdom, France, and the United States, and rational OLMU is permitted in these countries (6). As per the “Amendments to the Indian Medical Council Act,” the off-label prescription is banned in India (7). However, according to drug regulations in Pakistan, there is no clear explanation of off-label prescription practices.

The scarcity of controlled clinical trials in vulnerable patients is the key factor for the common practice of OLMU. Many physicians consider that OLMU has a significant role in therapeutic practice representing the optimum way to utilize that treatment and OLMU is frequently required when treating certain patients, such as those whose symptoms have proved resistant to a variety of therapeutic options (8). OLMU may be beneficial in some cases because it provides evidence-based therapeutic alternatives to patients who have no other options, such as in situations where no authorized medications exist or for patients who have used the typical empiric treatment. Though OLMU may be therapeutically justifiable in some cases, it is connected with a variety of safety and ethical concerns (8). OLMU can affect the patient wellbeing in circumstances when a benefit-risk ratio has not been completely established. This is mostly because OLMU is generally not evaluated by regulators, guidance developers, or even healthcare legislators (9). When carefully evaluated, certain regularly practiced OLMU have likewise been revealed to be either hazardous or useless (10). The expert consensus that for safe use of OLMU they must be based on supportive evidence and being approved by the ethics or formulary committee (11). Besides the informed consent must be obtained from the patient and adverse drug reactions (ADRs) should be monitored and regularly updated in a database of off-label drugs (12).

Previous studies shown that the healthcare experts emphasize that OLMU has a significant role in therapeutic practice, but they likewise acknowledge that using an OLMU might increase the risk of claims if a patient has unfavorable or severe adverse responses (13, 14). In Pakistan, being a developing country, the economic burden of OLMU could further deteriorates the situation and therefore it is imperative to study this issue in the local context. Several studies have been conducted on the health professionals' insight regarding OLMU in different medical conditions; nevertheless, to the best of our knowledge, no such work has been published regarding the OLMU in the treatment of female reproductive health issues (FRHIs) (8, 9, 12, 14). Hence, the purpose of the current study was to assess gynecologists/obstetricians' knowledge, attitude and practice toward OLMU in the treatment of FRHIs.

## Methodology

### Study Design and Setting

A survey based quantitative cross-sectional study was conducted in September and October 2021, at five tertiary care hospitals (two public and three private sector), different clinics and maternity homes in a metropolitan city of Karachi, Pakistan. The target population was gynecologists, obstetricians and physicians/residents working in the ob/gyn department in various hospitals and clinical settings of Karachi and willing to participate in the study. The questionnaire were distributed through email or personal contacts and the respondents were invited to complete the survey form. A reminder email was sent to those who had not responded after a week to get the maximum response rate.

### Ethical Approval

The study was conducted as per the recommendations of the “Declaration of Helsinki” and the approval was obtained from the Ethical Review Committee of Sohail University with the protocol # 000125/21. The written consent was obtained from the respondents before the study and the goals of the study were explained to them.

### Sampling Technique

The study sample size was calculated by G Power software, version 3.0.10 (15), by using Chi-square goodness of fit test with an effect size of 0.2, alpha error 0.05, and power of test 0.8 (8); the calculated sample size was found to be 197. The convenient and snowball sampling techniques were used to select the respondents.

### Research Instrument Development and Piloting

The questionnaire was derived from a review of prior studies (3, 12, 16–18). In addition to the demographic information the study questionnaire had four sections:

- The first section had five closed ended questions with “true,” “false,” and “do not know” options designed to evaluate the respondents' awareness of OLMU in the treatment of FRHIs, their concerns about its safety and efficacy, and the possibility of ADRs when taking OLMU.
- The second section consists of 15 closed ended questions designed to assess respondents' current practice of prescribing different categories of OLMU in the treatment of FRHIs, their proclivity to prescribe OLMU and perceived barriers. The items were scored on a 5-point Likert scale, ranging from strongly agree = 5 to strongly disagree = 1.
- The third section consists of five closed ended questions with “yes” or “no” options about their practice of obtaining informed, written/verbal agreement from patients before prescribing OLMU in the treatment of FRHIs.
- The fourth section consists of five closed ended questions enquiring about the respondents' knowledge sources and examining their ideas for minimizing such practices.

The face validity and content validity of the questionnaire was subjected by three gynecologists working in Dow University Hospital. As per the experts' recommendations, minor adjustments were made to the final version of the questionnaire. The piloting of the research questionnaire was conducted on 20 gynecologists and the research questionnaire was validated. The Cronbach-alpha test was conducted to verify the questionnaire's reliability and internal consistency. The dependability coefficient was 0.733, which was within an acceptable range.

### Data Collection and Analysis

The data entry and analysis of responses collected were done by the Statistical Package for Social Sciences v.20.0 (SPSS; Chicago, IL). The demographic data of the respondents were illustrated as percentages and frequencies. The Shapiro-Wilk test was performed to establish the normality of the variables. Pearson-correlation coefficients and analysis of variance were employed to identify the relationship between the independent variables and the responses considering *p*-values <0.05 as statistically significant.

## Results

### Demographic Information

In the current research, 152 gynecologists, obstetricians and physicians/residents completed the survey; hence, the overall response rate was 77.1%. Only *n* = 9 (5.9%) were males and *n* = 143 (94.0%) were females. The mean age of the study respondents was 36.1 ± 7.7 years with *n* = 85 (55.9%) were working in primary patient care. The respondents *n* = 52 (34.2%) whereas *n* = 48 (31.5%) were having a working experience of 6–10 and 1–5 years, respectively. Table 1 depicted the detailed demographics of the study population.

**Table 1:** Respondents' demographic characteristics.

**Baseline characteristics**	**Frequency (%)**
**Gender**	
Male	9 (5.9)
Female	143 (94.0)
**Working organization**	
Private	112 (73.6)
Public	40 (26.3)
**Working experience**	
<1 year	13 (8.5)
1–5 years	48 (31.5)
6–10 years	52 (34.2)
11–20 years	33 (21.7)
More than 20 years	6 (3.9)
**Practice area**	
Primary patient care	85 (55.9)
Secondary patient care	31 (20.3)
Tertiary patient care	36 (23.6)

### Respondents' Knowledge and Attitude Toward OLMU

**Figure 1:**
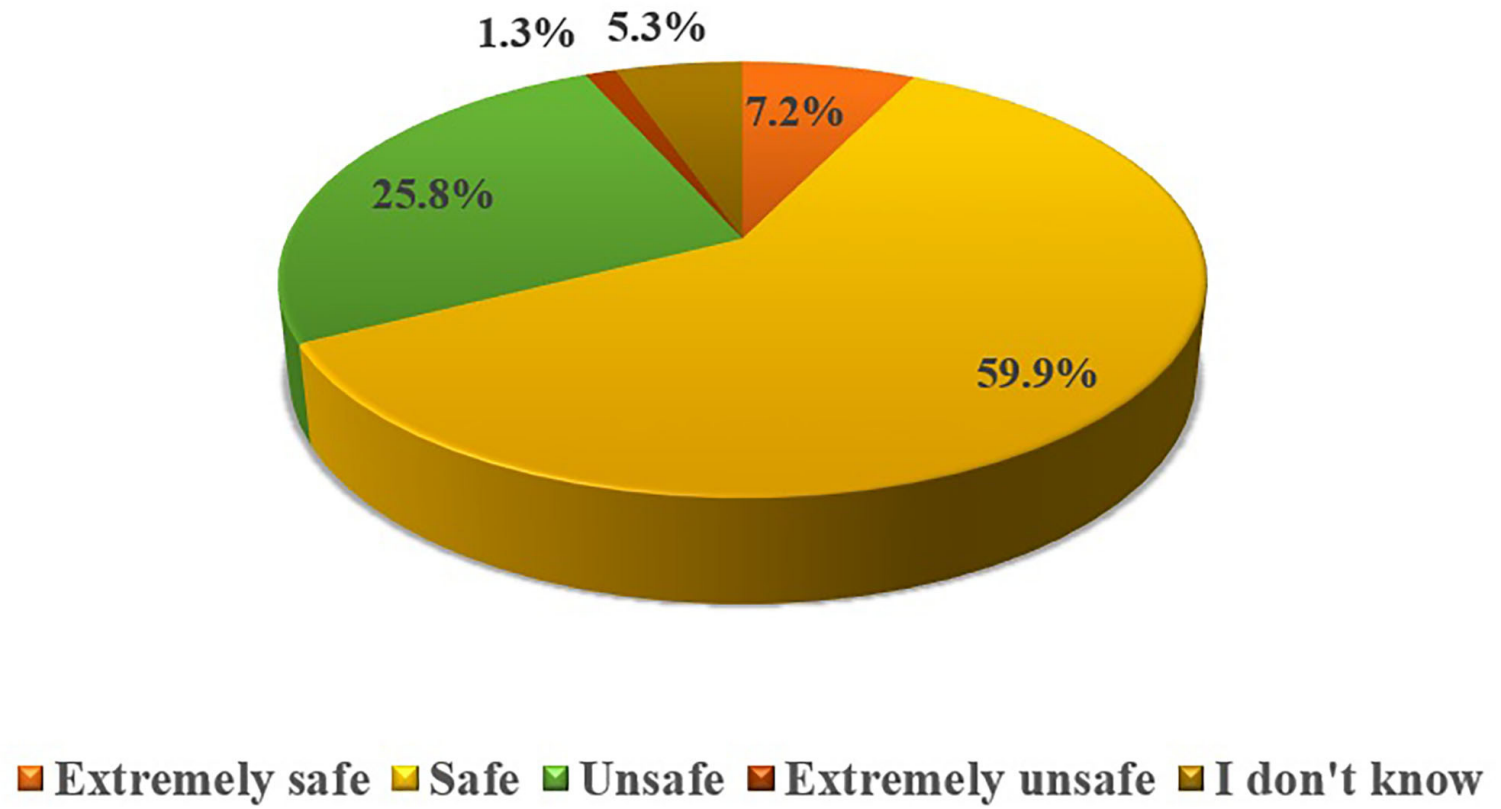
Respondents' attitude toward OLMU safety in the treatment of female reproductive health issues.

The respondents *n* = 120 (78.9%) were well-familiar with the concept of OLMU and *n* = 70 (46.1%) had heard about the OLMU during their professional life. However, their knowledge was found to be statistically significant with their practice area (*p* = 0.0001). On inquiring about the safety of OLMU, around 60% of respondents considered them safe if used with appropriate scientific evidence (Figure 1). The respondents *n* = 108 (71.1%) consider that there is no meaningful difference between the on-label and OLMU in terms of quality, safety and efficacy. This opinion was varying significantly with the experience (*p* = 0.017) and practice area (*p* = 0.025) of respondents. Around 70% of the respondents showed their concerns when prescribing OLMU about its safety and efficacy in the treatment of female infertility and consider that some OLMU could increase the likelihood of ADRs. However, around 85% stated that have not observed any ADR when using OLMU in their practice.

### Respondents' Perceived Reasons of Practicing OLMU

**Figure 2:**
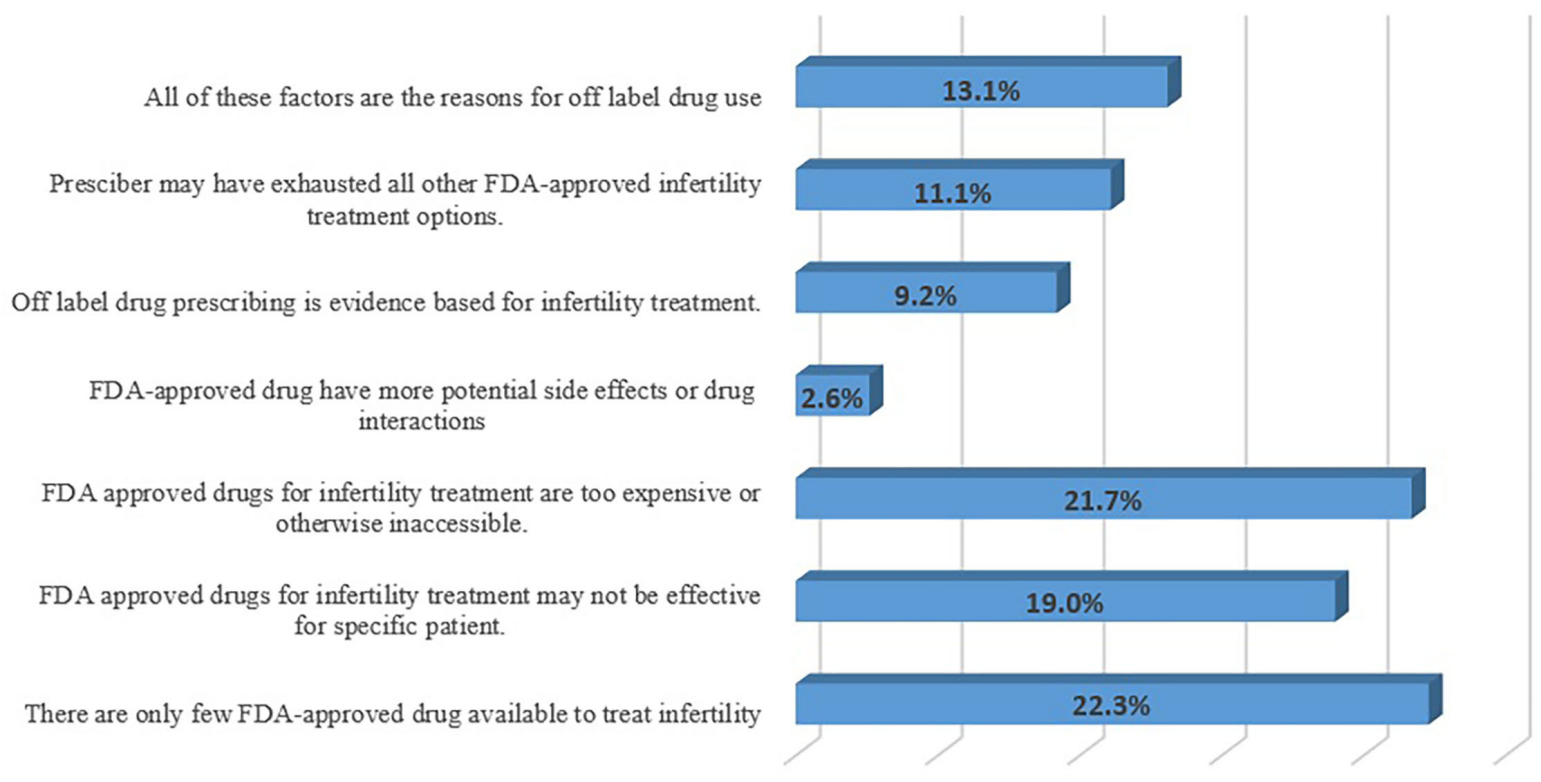
Respondents' perceived major reasons for increased use of OLMU.

More than half of the respondents (*n* = 84; 55.2%) consider that the OLMU allows for innovation in healthcare practice, especially when recognized therapies have failed; however, the response was varied with the working experience (CI 2.21–2.73; *p* = 0.003) and practice area (CI 2.28–3.52; *p* = 0.005) of respondents. The respondents (*n* = 80; 52.6%) think that the OLMU provides clinicians with early access to potentially useful drugs, as well as the ability for professionals to adopt new procedures based on developing evidence; however, the response was varying significantly with the working organization of respondents (CI 2.42–2.93; *p* = 0.001). The respondents perceived major reasons for increased use of OLMU in the treatment of FRHIs were availability of only a few FDA-approved drugs (*n* = 34; 22.3%) and FDA-approved drugs are too expensive or otherwise inaccessible (*n* = 33; 21.7%) (Figure 2). The respondents (*n* = 97; 63.8%) consider that the symptoms frequently cross over from one illness state to the next, prompting clinicians to utilize drugs for unapproved gynecological indications; the response varying significantly by the working organization (CI 2.12–2.65; *p* = 0.05) and practice area of the respondents (CI 2.17–3.24; *p* = 0.004).

### Commonly Used Categories of OLMU in Practice of Respondents

**Figure 3:**
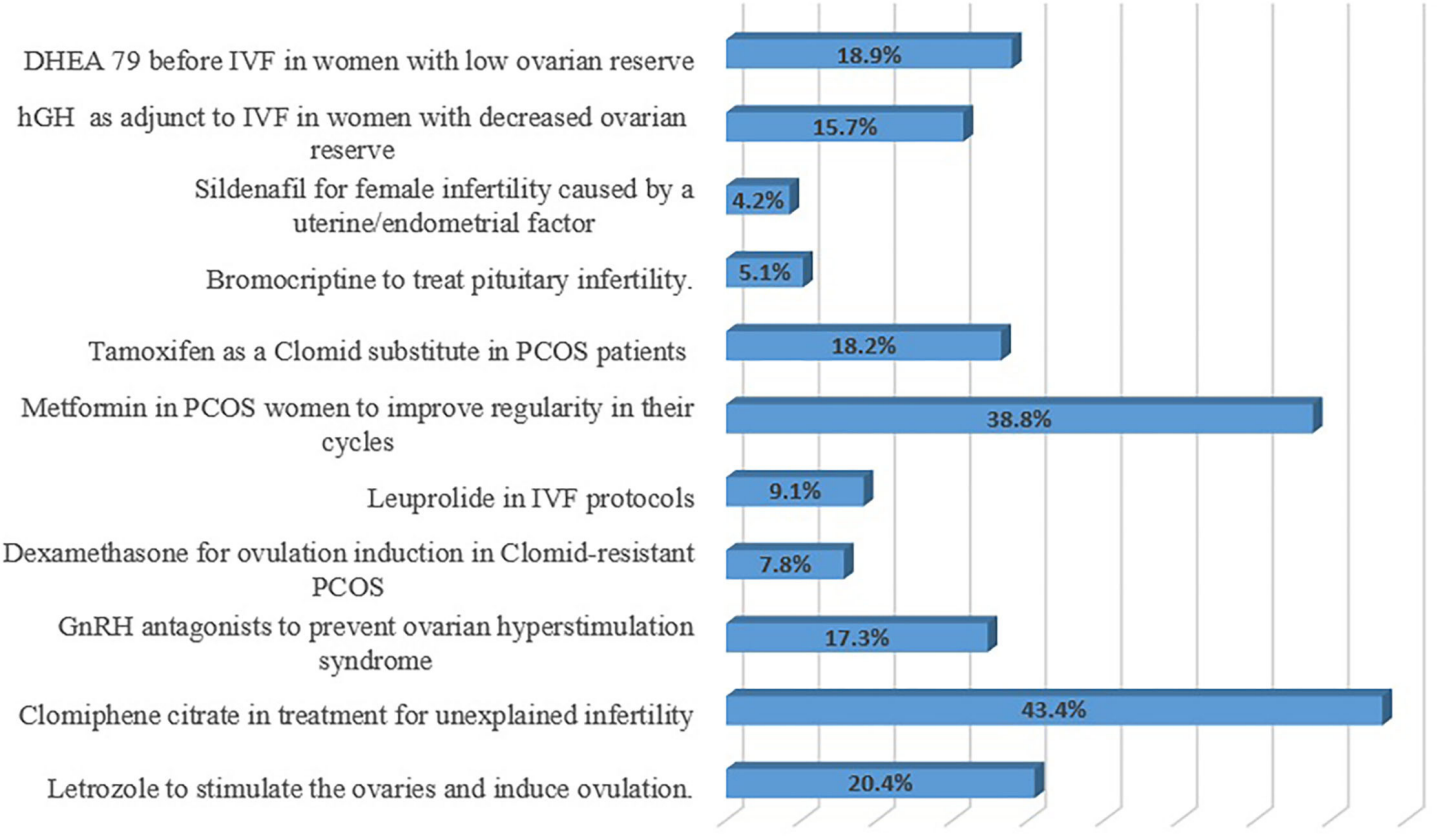
Respondents' most commonly used category of OLMU in their practice. GnRH, Gonadotropin-releasing hormone; IVF, *In vitro* fertilization; PCOS, Polycystic ovary syndrome; hGH, Human growth hormone; DHEA, Dehydroepiandrosterone.

**Figure 4:**
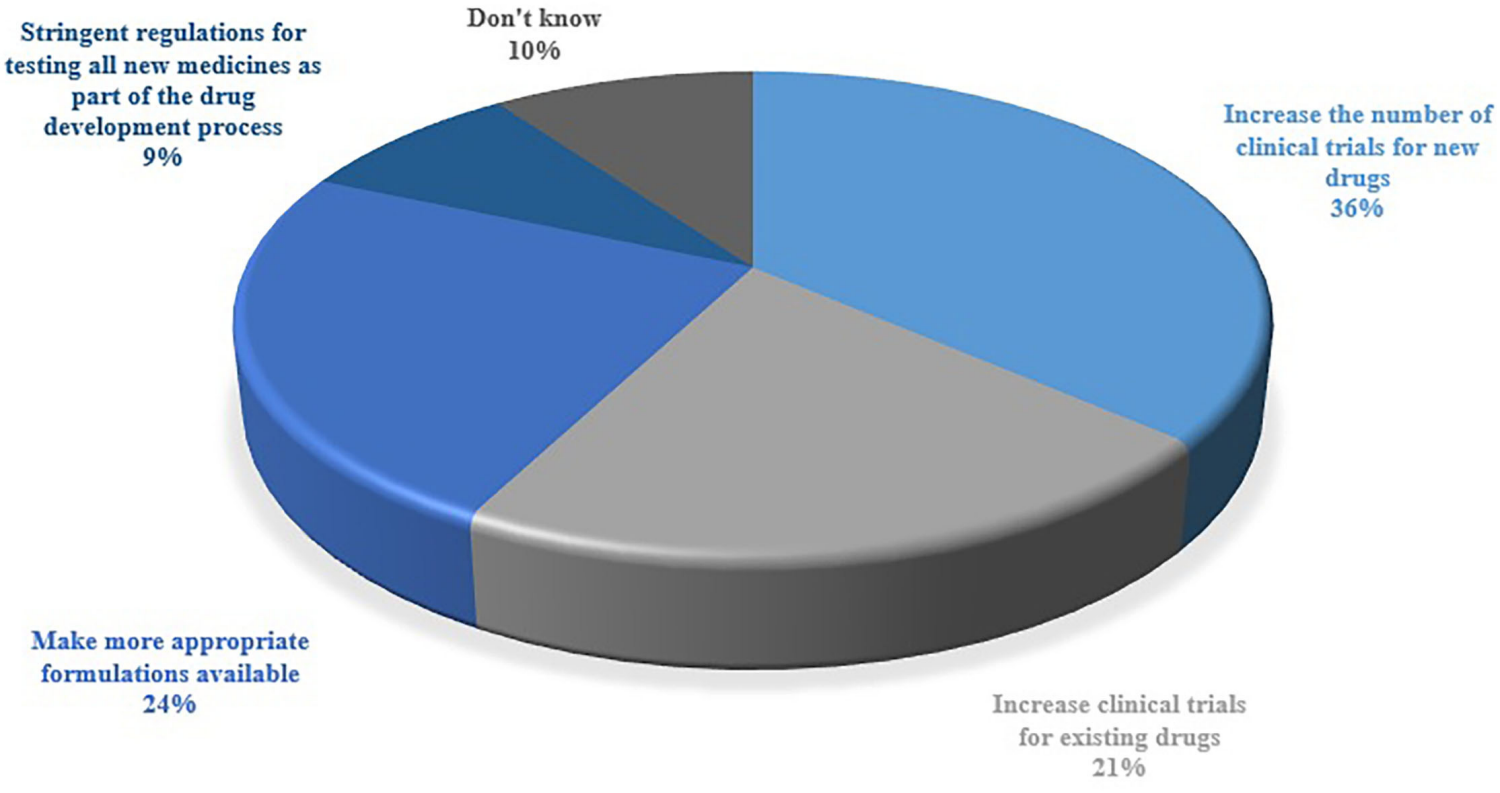
Respondents' perceived beneficial factors in reducing the OLMU for infertility treatment.

The respondents were inquired about the category of OLMU that is most commonly used in their practice (Figure 3). The majorly reported OLMU by the respondents were clomiphene citrate as an infertility treatment in males (*n* = 66; 43.4%), metformin to improve cycle regularity in females with polycystic ovary syndrome (PCOS) (*n* = 59; 38.8%) and letrozole to induce ovulation (*n* = 31; 20.4%). The majorly stated off-label prescribing were at a different dose than the one it is approved to treat (*n* = 95; 62.5%) and at different indications than the one it is approved to treat (*n* = 89; 58.5%). Figure 4 depicted the factors which can be more beneficial in reducing the OLMU. The majorly reported factors were increasing the number of clinical trials for new drugs in infertility patients (*n* = 55; 36.1%) and making more appropriate formulations available for infertility patients (*n* = 37; 24.3%).

### Respondents' Practice of Informing Their Patients About OLMU

Around 40% of the respondents informed their patients when prescribing an OLMU whereas more than 75% did not request informed, written consent/verbal consent from patient or attendant before prescribing OLMU. The respondents (*n* = 91; 59.9%) think that physician who is prescribing the off-label has the main responsibility to inform the patient about OLMU to treat them. The majority of the respondents (*n* = 104; 68.4%) agreed that it would needlessly worry the patient if we told them that medicine that she was being treated with was used in an off-label manner. The respondents (*n* = 95; 62.5%) agreed that the benefits associated with OLMU outweigh the associated risk whereas (*n* = 91; 59.6%) consider that OLMU has the potential to become widely accepted in clinical practice and to become the standard therapy for treating different female reproductive health issues.

### Respondents' Perceived Barriers for OLMU and Their Reliable Sources of Information

**Figure 5:**
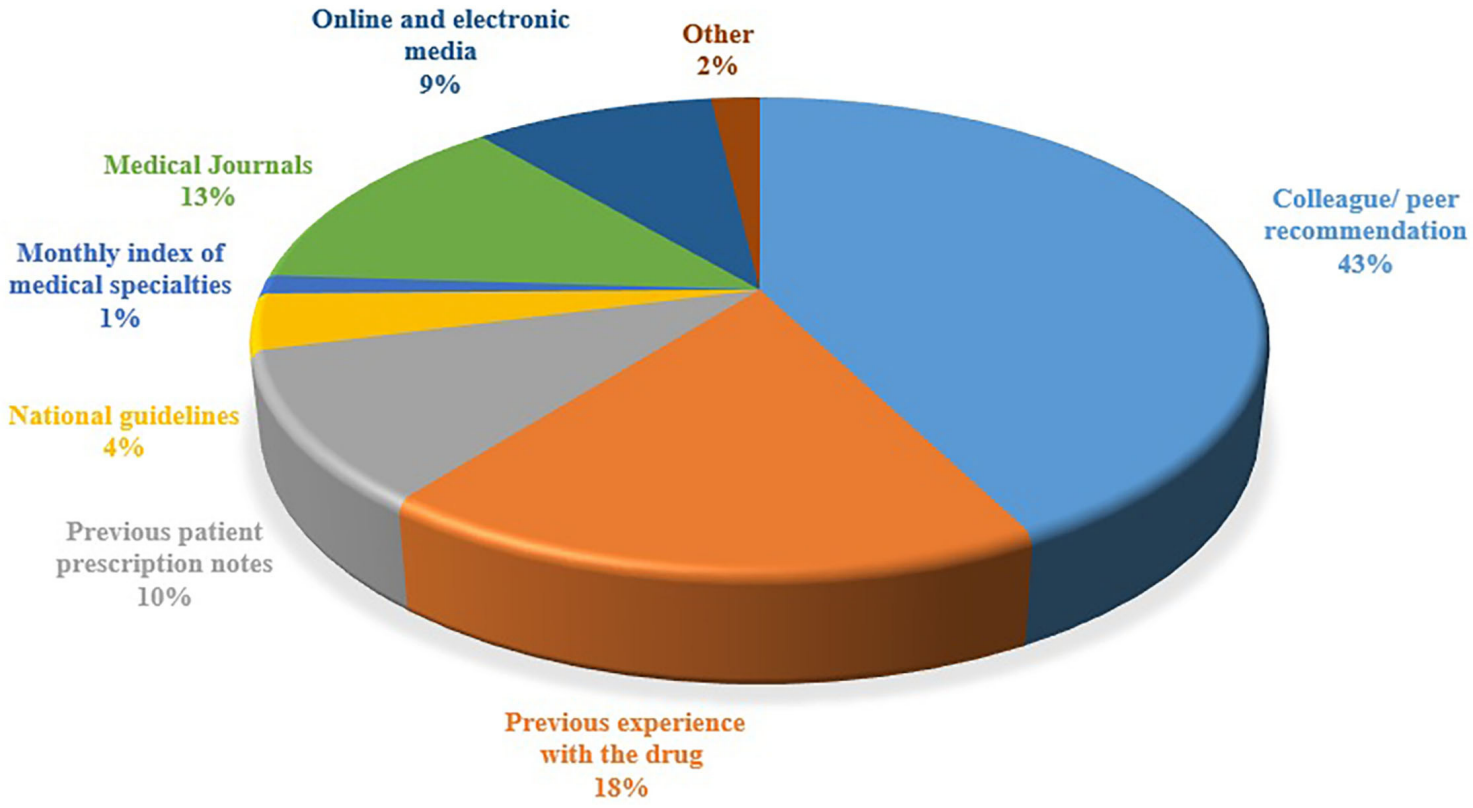
Respondents' major sources of information.

The majority of the respondents (*n* = 115; 75.6%) deemed that the healthcare professionals risk legal liability if they use OLMU in patient care, especially if the patient develops an ADR. It was reported by the majority of the respondents (*n* = 95; 62.5%) that they do not follow any guidelines or regulations for OLMU in their working hospital; however, the response was found to be statistically associated with the working organization (CI 2.14–2.93; *p* = 0.037) and practice area (CI 2.85–4.32; *p* = 0.0001) of respondents. The respondents' perceived majorly reported ADRs of fertility drugs were ectopic pregnancy (*n* = 29; 19%), Ovarian Hyperstimulation Syndrome (OHSS) (*n* = 25; 16.4%) and having multiples (*n* = 22; 14.5%). The majority of the respondents (*n* = 102; 67.1%) stated that they were not familiar with how to cope with the side effects of fertility drugs. The respondents' major sources of information were colleague/peer recommendation (43%), previous experience with the drug (18%) and medical journals (13%) (Figure 5).

## Discussion

The world's population is facing enormous challenges due to reproductive issues; with an obvious concern of fertility issues and a declining birth rate (3). Numerous endogenous and exogenous variables have an impact on female reproductive health. Many FRHIs are becoming more prevalent due to the increased release into the environment of industrial chemicals with analogous properties, and many of these substances have been documented as entering the human body via urine or blood (1, 2). As far as we possibly know, this is the first study conducted in a Pakistani setting on gynecologists/ obstetricians' knowledge, attitudes, and practices related to the use of OLMU. The outcomes of the current study revealed considerable understanding of OLMU among the respondents as they were found to be well-aware with the concept. Other studies indicated the parallel findings depicting the major reasons for awareness of OLMU among healthcare professionals were their post-graduate and undergraduate teaching and practical exposure (18, 19). The majorly reported OLMU by the respondents were clomiphene citrate in unexplained infertility to encourage the growth of multiple follicles (*n* = 66; 43.4%), metformin to improve cycle regularity in women with PCOS (*n* = 59; 38.8%) and letrozole to induce ovulation (*n* = 31; 20.4%). Clomiphene citrate has both estrogenic and antiestrogenic characteristics and is frequently used off-label for the treatment of unexplained infertility, both alone and in conjunction with intrauterine insemination (IUI) (20). Femara, or letrozole, was not intended to be a fertility medication. It is, in reality, a breast cancer medication (20). It is, however, increasingly often used off-label to address ovulation problems. According to statistics given at the 34th Annual Meeting of the European Society of Human Reproduction and Embryology (ESHRE), a multinational, cross-sectional online survey indicated that 75.5% of physicians suggested OLMU of androgens or letrozole to infertile women (21). Nearly half of fertility experts who did not suggest androgens cited a lack of scientific evidence as to the reason for their therapeutic decision (21). Metformin is one of the most often utilized off-label drugs for PCOS causing infertility issues. Metformin has quickly move up from its early rational use for the treatment of ovulation induction in PCOS to one of the most sought drugs for the condition's management (22). Regardless of the vast number of complete investigations, there is great disagreement among experts with little definitive data on its usefulness in PCOS (23). In our study, we found a similar trend of increased off-label prescribing of both clomiphene and metformin for various reproductive therapies.

According to the current findings, respondents knew that practice of OLMU may compromise safety of patient in certain clinical situations where a progressive risk-benefit ratio isn't completely well-recognized. Around 70% of the respondents showed their concerns when prescribing OLMU about its safety and efficacy in the treatment of FRHIs and consider that some OLMU could increase the likelihood of ADRs. Similar trend was observed in other studies revealing the concern of healthcare professionals toward the safety and efficacy issues of OLMU (16, 24, 25). There is not any clear description of the right to prescribe OLMU anywhere in the globe. The Drugs and Healthcare Products Regulatory Agency in the United Kingdom allows physicians, dentists, pharmacists and independent nurses to prescribe OLMU (26). In Pakistan only physicians have prescription rights and by constraining the prescribing practice of OLMU by the physicians we can prevent the misuse of OLMU. Healthcare professionals are legally restricted to inform patients toward the jeopardies involved in using OLMU (24, 25). The element the there is a lack of data available for using the OLMU must be reflected as a likelihood of hazards to the patient. The physicians should observe legitimate practices that expect them “to obtain informed consent from a person before performing a test or stating a treatment—particularly a treatment that involves some uncertainty”. In the present study, the majority of the respondents (75.6%) deemed that the healthcare professionals risk legal liability if they use OLMU in patient care, especially if the patient develops an ADR. Patients, particularly females in less developed nations, face challenges such as a lack of knowledge and information on different health issues, period poverty or stigma, a lack of access to treatments, and gender inequities (1). It is anticipated that healthcare professionals should inform patients regarding OLMU, however this doesn't appear to be the situation in the present study. It was worrying that around 40% of the respondents informed their patients when prescribing an OLMU whereas more than 75% did not request informed, written / verbal consent from patient or attendant before OLMU. Shakeel et al. reported that the majority of the responders did not notify the patient while prescribing an off-label (27). Varying approaches for informed consent have been proposed in Australia based on different degrees of evidence in off-label medication (28).

In the current study, it was reported by the majority of the respondents (62.5%) that they do not follow any guidelines or regulations for OLMU in their working hospital; however, the response was varied significantly with the working organization (CI 2.14–2.93; *p* = 0.037) and practice area (CI 2.85–4.32; *p* = 0.0001) of respondents. Another study reported that the respondents (74 %) stated that OLMU is not permitted by law, and they (88%) also deemed that there should be clear norms and procedures for OLMU (3). A lack of clear guidelines can lead to uncertainty in physicians' practices and patient discontent. Only by instituting clear norms, direction, and access to scientific knowledge could uncertainty and confusion be avoided (29). The hospital's pharmacy and therapeutics (P&T) committee should be viewed as the arbitrator of institutional policy regarding OLMU, and its decisions should be driven by scientific evidence. When considering OLMU, supporting safety and data must be thoroughly assessed, and a risk-benefit analysis must be undertaken, especially when FDA-approved alternatives are available. Before permitting OLMU therapy, a systematic approach for evaluating scientific evidence should be implemented (30).

The respondents in the present study reported ectopic pregnancy, OHSS and having multiples as the major ADRs of fertility drugs and stated that they do not know how to cope with those side effects. The drugs used to treat fertility issues have their own set of negative consequences (31). Although many of these side effects are harmless and self-limiting, others, particularly those related to gonadotropins, may be severe. Clomiphene has been associated with hot flushes, multiple gestations, visual difficulties, abnormal cervical mucus, and luteal phase deficit (32). OHSS is described by enlarged ovaries and fluid buildup in the abdomen following gonadotropin stimulation and ovulation. A moderate form could occurs in 10–20% of cycles, causing some pain however typically resolving quickly and without consequences (33). Having a trustworthy source of health information is critical for developing a solid theoretical foundation, especially in light of the current internet and social media revolution, which raises numerous concerns about the public's health (34). Colleague/ peer recommendation, previous experience with the drug and medical journals were the respondents' major sources of information for fertility treatment in the present study. Similar outcomes were reported by another study depicting peer recommendations as the major source of health information and prescribing practice (34).

Hence the present study found that respondents were well-familiar with the practice of OLMU in the treatment of FRHIs; yet, they expressed their concern toward decreasing such practice. The findings of our study are supplemented by one more study from Pakistan that shows a comparable pattern of increasing OLMU prescribing trends in clinical settings (16). OLMU is a global thought-provoking problem that has not been explored earlier in Pakistan. Appropriate policies tailored to the local context must be developed and implemented by health establishments to evade the problems associated with inapt prescribing in patients.

The strength of the study lies with the fact that as far as we possibly know there is no such investigation on OLMU focusing particularly on FRHIs reported earlier in Pakistan. This was the first study that was conducted in Pakistan to evaluate the practice of gynecologists/obstetricians in this context. The limitation of this study is the small sample size and the study was conducted with gynecologists/obstetricians in only few clinics and hospitals of single city Karachi; hence the findings are not generalizable to gynecologists/obstetricians in all cities of Pakistan and other countries. Besides, due to the nature of the current study, we were only able to explore the topic superficially, and additional in-depth research, such as investigating off-label practice throughout the country and strategies for overcoming hurdles, might be beneficial in the future.

## Impact on Practice

- Further extensive training of health care professionals on off-label issues; for instance, the collective utilization of contemporary sources of information, coupled with informal specialized networks is desired.
- There is a need of improved dissemination of evidences for off-label indications among healthcare team.
- It ought to be pursued that health experts should inform patients regarding OLMU, however this doesn't appear to be the situation in current practice.

## Conclusions

The present outcomes revealed that the practice of OLMU in the treatment of FRHIs was well-observed in the study. However, the respondents were concerned about decreasing such practices by being involved in collective decision-making procedures, and were inclined to accept initiatives aimed at ensuring drug safety in patients. Policies should be established tailored to the local context to analyze OLMU that negotiates patient safety or signifies an improvident medication use.

## Data Availability Statement

The original contributions presented in the study are included in the article/supplementary material, further inquiries can be directed to the corresponding author/s.

## Ethics Statement

The study was conducted as per the recommendations of the Declaration of Helsinki and the approval was obtained from the Ethical Review Committee of Sohail University with protocol # 000125/21. The written consent was obtained from the respondents before the study and the goals of the study were explained to them. The patients/participants provided their written informed consent to participate in this study.

## Author Contributions

SSh, WI, AQ, SN, FB, SSi, HR, and AR made substantial contributions to the conception and design of the study and the analysis and interpretation of the data. SSh and AR made substantial contributions to the analysis and interpretation of the data. All authors drafted the work or revised it critically for important intellectual content, reviewed, critiqued, and approved the final version submitted for publication.

## Conflict of Interest

The authors declare that the research was conducted in the absence of any commercial or financial relationships that could be construed as a potential conflict of interest. The reviewer SI declared a past co-authorship with the author HR to the handling editor.

## Publisher's Note

All claims expressed in this article are solely those of the authors and do not necessarily represent those of their affiliated organizations, or those of the publisher, the editors and the reviewers. Any product that may be evaluated in this article, or claim that may be made by its manufacturer, is not guaranteed or endorsed by the publisher.
